# Lower Cognitive Function in People Aged 60 to 69 Years Is Associated With Future Stroke

**DOI:** 10.1161/JAHA.125.048423

**Published:** 2026-09-30

**Authors:** Alice Askemyr, Rani Basna, Sölve Elmståhl, Marieclaire Overton

**Affiliations:** ^1^ Division of Geriatric Medicine, Department of Clinical Sciences in Malmö Lund University, Skåne University Hospital Malmö Sweden

**Keywords:** cognitive domains, global cognition, prestroke cognition, stroke, Cognitive Impairment, Cerebrovascular Disease/Stroke, Aging, Risk Factors

## Abstract

**Background:**

The association between impairment across cognitive domains and incident stroke has not been thoroughly investigated. This study examined the risk of first‐time stroke among older adults with lower cognitive function in different cognitive domains and how associations vary by age.

**Methods:**

This longitudinal cohort study used baseline data from GÅS (Good Aging in Skåne) and registry data from the Swedish Board of Health and Welfare. Exclusion criteria were previous stroke or transient ischemic attack, baseline dementia, or no cognitive data. The participants (n=4912) were divided into tertiles on the basis of performance on cognitive tests representing multiple cognitive domains. Cox regression was used to estimate the risk of stroke for the low tertile of cognitive function compared with the high tertile. Age‐stratified analysis was performed for participants aged 60 to 69, 70 to 79, and ≥80 years.

**Results:**

The studied population consisted of 2203 men, 3094 aged 60 to 69 years, 494 aged 70 to 79 years, and 1184 aged ≥80 years. During the average follow‐up time of 12.3 years, 572 of the 4912 participants had a stroke. In the fully adjusted models, participants in the low tertile of perceptual speed had higher risk of stroke (hazard ratio [HR], 1.40 [95% CI, 1.13–1.72]). In age‐stratified analysis, higher risk of stroke was observed for participants aged 60 to 69 years in the low tertile of global cognition (HR, 1.86 [95% CI, 1.27–2.69]), episodic memory (HR, 1.68 [95% CI, 1.21–2.33]), and perceptual speed (HR, 1.66 [95% CI, 1.19–2.32]).

**Conclusions:**

Lower cognitive functioning in global cognition, episodic memory and perceptual speed in people aged 60 to 69 years are associated with an increased risk of future stroke.

**Nonstandard Abbreviation and Acronym**
GÅS: Good Aging in Skåne (Gott Åldrande i Skåne)

**Research Perspective**

**What Is New?**

- The findings of this study raise questions about whether low cognitive function in younger older adults can serve as a marker for increased stroke risk.

**What Question Should Be Addressed Next?**

- Subsequent studies in younger cohorts should investigate the potential use of cognitive test performance in combination with biomarkers as indicators of future stroke risk.
- Future magnetic resonance (MR) studies should examine associations between lower cognitive performance, preexisting cerebrovascular damage, and subsequent stroke.

In 2021, stroke was the third‐leading cause of death worldwide.^1^ The incidence of stroke and cognitive impairment increases with age, and several shared risk factors have been suggested, including hypertension, diabetes, physical inactivity, smoking, and excessive alcohol use.^2^, ^3^, ^4^, ^5^ In addition, cognitive impairment has been considered an expression of preexisting cerebrovascular damage, such as silent brain infarcts, leukoaraiosis, and cerebral microbleeds.^6^ These conditions are furthermore linked to an increased risk of stroke.^7^, ^8^, ^9^ Cognitive impairment may therefore serve as an important indicator of the need for preventive measures against further cerebrovascular disease.

Two meta‐analyses established a higher relative risk of stroke for those with cognitive impairment.^10^, ^11^ However, studies considering cognitive impairment across different domains are few.^12^, ^13^, ^14^, ^15^, ^16^, ^17^, ^18^, ^19^ Studies investigating episodic memory have shown various results, where some indicated higher risk of stroke for those with impaired memory,^14^, ^15^, ^16^ while others did not.^12^, ^13^, ^17^, ^18^ Similar inconsistent results can be seen for executive function,^16^, ^17^, ^18^, ^19^ language,^12^, ^13^, ^16^, ^18^ and perceptual speed.^12^, ^13^, ^16^, ^18^, ^19^ Therefore, more studies are warranted. Although the risk of stroke among individuals with cognitive impairment may vary with age, this stratified risk has not been thoroughly investigated.^20^ Furthermore, previous studies have reported sex differences in the associations between major stroke risk factors and incident stroke.^21^ Thus, potential sex differences in the relationship between cognitive impairment and incident stroke warrant consideration.

Accordingly, the aim of this study was to investigate the risk of first‐time stroke among older adults with lower cognitive functioning in different cognitive domains. It further aimed to investigate how associations vary across age groups.

## Methods

### Data Availability

The data that support the findings of this study, as well as analytic models, are available from the corresponding author upon reasonable request.

### Study Design and Participants

A prospective cohort study design was used. The majority of the data were extracted from the longitudinal, general population study GÅS (Good Aging in Skåne [Gott Åldrande i Skåne]), which is included in the Swedish National Study on Aging and Care.^22^, ^23^ The Swedish population register was used to randomly select older adults across 5 municipalities in Skåne County in southern Sweden.

The first 3 waves of participants were included in this study. The first wave (2001) of participants consisted of men and women aged 60 to 99 years. New participants aged 60 and 81 years were invited in 2007 (wave 2) and 2012 (wave 3). The participation rate ranged from 60% to 70% (data on nonparticipation characteristics are not available). For this study, follow‐up lasted until 1 of the following occurred: (1) first‐time stroke, (2) death, or (3) end of registry data (December 31, 2023). Date of death was obtained from the Swedish Cause of Death Register provided by the Swedish Board of Health and Welfare.

The same standardized physiological, psychological, and medical examinations were carried out at all 3 waves. In addition, participants were asked to fill out self‐rate questionnaires. Information was also retrieved from medical records. A total of 5804 participants were examined at baseline. Exclusion criteria for the current study were participants with no cognitive data, dementia, previous stroke, or transient ischemic attack (TIA) at baseline. Participants with dementia or a history of stroke at baseline were excluded, as they would likely be overrepresented in the low cognitive function group compared with higher‐functioning groups. This exclusion was necessary because the study aimed to investigate first‐time stroke and to minimize reverse causation arising from poststroke cognitive impairment.^24^ TIA cases before baseline were excluded on the basis that a TIA effects risk of stroke events^25^, ^26^ as well as the effect of TIA on cognition.^27^

### Cognitive Assessment

The procedure of cognitive testing has been described in detail elsewhere.^28^ Standardized testing methods were implied, reducing issues such as interrater reliability.^29^ Episodic memory was measured through immediate recall and recognition of words. The digit cancellation test and the pattern comparison test were used to measure perceptual speed, while word fluency in naming animals and occupations was used for measuring word fluency categories.^28^ The tests for episodic memory, perceptual speed, and word fluency categories have been deemed to be reliable and valid to represent the domain in an earlier study.^30^ In addition, selected components of executive function were measured by digit span backwards test and a shortened version of the Trail Making Test B, which capture working memory and cognitive flexibility.^28^

A *Z* score for each test score was generated for the participants. A composite score for each cognitive domain, that is, the mean value of the *Z* scores for representative tests, was calculated. For participants who had completed tests of ≥2 cognitive domains, global cognition was represented by a composite score of the completed tests, that is, the mean value of the *Z* scores of all available tests.^31^ The cognitive domains were then further divided into age‐stratified tertiles, representing low, middle, and high performance. The low tertile represented participants with lower cognitive function.

### Dementia and TIA Assessment

Dementia diagnosis was obtained from the examining physician, using the *Diagnostic and Statistical Manual of Mental Disorders, Fourth Edition* ^32^ for all‐cause dementia, and history of dementia from medical records. Complementary dementia diagnosis and TIA events were obtained from the Swedish National Patient Register of specialized in‐ and outpatient care provided by the Swedish Board of Health and Welfare.

### Outcome

Data on first‐time stroke events were extracted from the Swedish National Patient Register of specialized in‐ and outpatient care. Stroke events included brain hemorrhages classified under *International Classification of Diseases, Tenth Revision* (*ICD‐10*) I60 to I61, brain infarction classified under *ICD‐10* I63.0 to I63.5 and I63.7 to I63.9, as well as unspecified stroke classified under *ICD‐10* I64. TIA was not included as an outcome.

### Covariates

Age, sex, and years of education, in addition to smoking status, alcohol usage, and physical activity status, were self‐reported at baseline examination. To create the age‐stratified tertiles and to explore age associations in stratified analyses, age was categorized into 3 groups: participants aged 60 to 69, 70 to 79, and ≥80 years. However, in all adjusted analyses, age was included in its continuous form to reduce residual confounding. The total number of years of education was considered a continuous variable. Previous smokers and individuals who had never smoked were considered nonsmokers, while current smokers were considered smokers. Alcohol consumption was defined as either high (twice or more a week) or low to moderate (less than twice a week). Individuals engaging in regular or irregular physical activity were considered active, while individuals not taking part in any physical activity were considered inactive.

Body mass index was calculated as the measured weight/(length/100)^2^ (kg/m^2^). Blood pressure was measured 3 times sitting down in the upper left arm by an automatic sphygmomanometer, where the third recording was used here. If a third measurement was not available, the mean value of the first and second measurements was used.

Blood samples were drawn during the visit and non–high‐density lipoprotein cholesterol was calculated as the difference between total cholesterol and high‐density lipoprotein cholesterol. The use of antihypertensives, as well as the use of cholesterol‐lowering treatment, were self‐reported.

Comorbidities included diabetes types 1 and 2, atrial fibrillation including atrial flutter, and ischemic heart disease. These were reported (1) by detection by the physician, ECG, or self‐report; (2) by detection in medical records; and (3) by the in‐ and outpatient registers provided by the Swedish Board of Health and Welfare. Ischemic heart disease was considered if the participant had a diagnosis of angina pectoris, prior infarcts, chronic ischemic heart disease, or heart failure.

### Statistical Analysis

Baseline characteristics were compared across the tertiles of global cognition. ANOVA was used for numerical data. While inspection of residual Q‐Q plots indicated some deviation from normality in some covariates, ANOVA was still considered robust due to the large sample size with close to equal group sizes. Furthermore, while post hoc Brown–Forsythe tests for homogeneity of variance showed significant results, inspection of group SDs and variance ratio indicated minor differences. Hence, results of the F‐test were deemed robust and reported. Post hoc Welch ANOVAs were performed as a sensitivity analysis and yielded similar *P* values. For nominal and ordinal data, the χ^2^ test was used. Multicollinearity among covariates was analyzed using the variance inflation factor and generalized variance inflation factor. All values were <2.0, which indicated no significant multicollinearity. Multiple imputations were carried through the covariates, where missing data ranged between 0.2% and 3.7%.

The risk of stroke for the tertiles of each cognitive domain was calculated with Cox proportional hazards regression analysis and pooled results were described in hazard ratios (HRs) with 95% CIs. To assess if lower cognitive functioning was associated with incident stroke, the highest tertile was used as reference and compared with the lowest and the middle tertile. In a sensitivity analysis, the middle tertile was used as the reference group. Furthermore, in post hoc sensitivity analysis, subdistribution HRs were estimated using Fine–Gray regression to account for the competing risk of death. For all regression analyses, a *P* for trend was obtained by treating the tertiles as a continuous variable, reflecting stepwise risk if dropping from a higher tertile to a lower. Minimum detectable HRs were calculated post hoc using the Schoenfield formula.

Three models were run to investigate associations in a stepwise manner, each including the variables of the former model(s): (1) demographic data (age, sex, and years of education), (2) lifestyle factors (physical activity, body mass index, smoking, and alcohol use), and (3) vascular factors (diabetes, systolic blood pressure, non–high‐density lipoprotein cholesterol, antihypertensive treatment, ischemic heart disease, atrial fibrillation or flutter, and cholesterol‐lowering treatment). Covariates were preselected on the basis of clinical rationale, with regards to literature of risk factors of stroke and cognitive impairment, as well as prior studies.^2^, ^3^, ^4^, ^13^, ^14^, ^15^, ^16^, ^17^, ^18^ Testing of the proportional hazard assumption using Schoenfeld residuals showed no violation.

Stratified analyses for age groups (60–69, 70–79, and ≥80 years) were conducted. To address potential sex differences in associated risk of stroke, a sensitivity analysis on the group with the largest number of participants (60–69 years) was performed. To facilitate result presentation and interpretation, only the results from the fully adjusted model (model 3) are presented in the tables. Unadjusted results are found in the Supplemental Material.

A *P* value of <0.05 was considered statistically significant. Benjamini–Hochberg false discovery rate correction was used to address multiple testing. The correction was applied to the *P* for trend in the primary and age‐stratified analyses. Sensitivity analyses were considered exploratory. R software version 2026.01.0 Build 392 (R Foundation for Statistical Computing, Vienna, Austria) was used for all analyses. The plot was graphed in R software using the survminer package.

The study is reported in accordance with the Strengthening the Reporting of Observational Studies in Epidemiology guidelines. The checklist is found in the Supplemental Material.

### Ethical Considerations

The GÅS study was approved by the Ethics Committee of Lund University (LU 744‐00). Written consent was gathered from all participants of the study. Information about the participants was stored securely and treated confidentially to prevent violation of integrity.

## Results

### Baseline Characteristics

A total of 5238 participants underwent baseline cognitive testing (Figure 1). Participants with prior TIA or stroke (n=217), dementia (n=93), or both (n=16) at baseline were excluded, leaving 4912 participants for analysis. Out of these, a global cognition score was calculated for 4772 participants.

**Figure 1:**
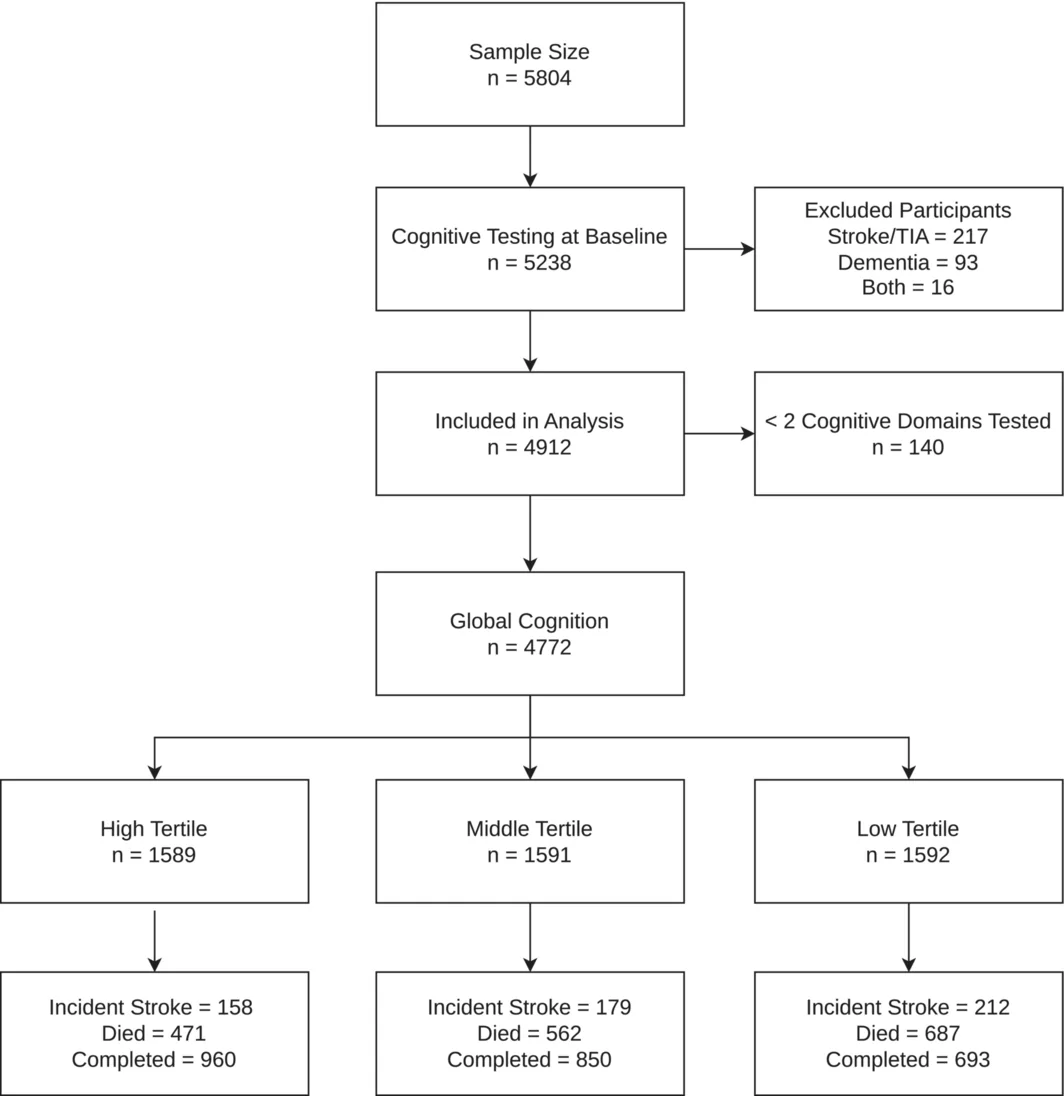
Flowchart showing participant inclusion and exclusion in the study. The proportion of participants who died were similar across cognitive domains. TIA indicates transient ischemic attack.

Baseline characteristics of the global cognition tertiles are shown in Table 1. The tertiles differed significantly in all described characteristics except age group, atrial fibrillation, cholesterol‐lowering treatment, and stroke subtypes. In total, there were 3094 participants aged 60 to 69 years, 494 aged 70 to 79 years, and 1184 aged ≥80 years. The studied population consisted of 2203 (46.2%) men.

**Table 1:** Baseline Characteristics of the Global Cognition Tertiles

Characteristics	Total (n=4772)	Tertiles of composite global cognitive score			*P* value
		High (n=1589)	Middle (n=1591)	Low (n=1592)	
Age, y, n (%)					1.000
60–69	3094 (64.8)	1031 (33.3)	1031 (33.3)	1032 (33.3)	
70–79	494 (10.3)	164 (33.2)	165 (33.4)	165 (33.4)	
≥80	1184 (24.8)	394 (33.3)	395 (33.4)	395 (33.4)	
Men, n (%)	2203 (46.2)	670 (30.4)	738 (33.5)	795 (36.1)	<0.001
Education, y, mean±SD	10.9±3.9	12.3±4.0	10.8±3.7	9.6±3.4	<0.001
Physical inactivity, n (%)	498 (10.4)	107 (21.5)	155 (31.1)	236 (47.4)	<0.001
Body mass index, kg/m^2^, mean±SD	27.1±3.9	26.8±4.2	27.0±4.5	27.5±4.8	<0.001
Current smoker, n (%)	803 (16.8)	216 (26.9)	266 (33.1)	321 (40.0)	<0.001
High alcohol consumption, n (%)	1129 (23.7)	521 (46.1)	381 (33.7)	227 (20.1)	<0.001
Diabetes, n (%)	367 (7.7)	100 (27.2)	110 (30.0)	157 (42.8)	<0.001
Systolic blood pressure, mm Hg, mean±SD	143.7±22.0	141.7±21.1	143.6±21.7	145.8±22.8	<0.001
Antihypertensive treatment, n (%)	1434 (30.1)	441 (30.8)	469 (32.7)	524 (36.5)	0.005
Non–high‐density lipoprotein cholesterol, mmol/L, mean±SD	4.3±1.1	4.2±1.1	4.3±1.1	4.4±1.1	0.001
Cholesterol‐lowering treatment, n (%)	749 (15.7)	257 (34.3)	235 (31.4)	257 (34.3)	0.082
Ischemic heart disease, n (%)	1018 (21.3)	274 (26.9)	344 (33.8)	400 (39.3)	<0.001
Atrial fibrillation, n (%)	269 (5.6)	83 (30.9)	85 (31.6)	101 (37.5)	0.321
Global cognition, mean±SD*	0.46±0.74	1.12±0.47	0.50±0.43	−0.23±0.56	<0.001
Episodic memory, mean±SD*	0.32±0.92	0.94±0.71	0.36±0.73	−0.34±0.84	<0.001
Perceptual speed, mean±SD*	0.85±1.07	1.54±0.89	0.86±0.85	0.12±0.97	<0.001
Selected components of executive function, mean±SD*	0.22±0.77	0.70±0.58	0.26±0.56	−0.32±0.78	<0.001
Verbal fluency, mean±SD*	0.50±1.06	1.30±0.89	0.51±0.79	−0.34±0.79	<0.001
Stroke, n (%)	549 (11.5)	158 (28.9)	179 (32.6)	212 (38.6)	0.011
Stroke subtypes					0.163
Ischemic stroke, n (%)	419 (8.9)	121 (28.9)	141 (33.7)	157 (37.5)	
Hemorrhagic stroke, n (%)	83 (1.7)	25 (30.1)	25 (30.1)	33 (39.6)	
Both, n (%)	2 (<0.1)	1 (50.0)	1 (50.0)	0 (0)	
Unspecified stroke, n (%)	45 (0.9)	12 (26.7)	12 (26.7)	21 (46.7)	

ANOVA was used for comparing numerical data, while χ^2^ test was used for categorical and ordinal data. Differences among the global cognition tertiles are noted by the *P* value. A *P* value<0.05 was considered statistically significant.

* Analysis performed on composites of *Z* scores of tests included in the cognitive domains.

### Primary Analyses

In Table 2, the adjusted HRs of incident stroke for the tertiles across cognitive domains are shown. During a mean follow‐up time of 12.3 years, 572 of the participants included sustained a first‐time stroke (Table S1). Minimum detectable HRs given the number of events are found in Table S2. After adjustments for demographic data, a higher risk of stroke was found for the low compared with the high tertile of global cognition, although with *P* for trend >0.05 (data not shown). In the fully adjusted models, a higher risk of stroke was found for the low tertile of perceptual speed in comparison with the high tertile (HR, 1.40 [95% CI, 1.13–1.72]). No significant associations were found in the fully adjusted models when comparing the low tertile with the high tertile for the whole sample for global cognition (HR, 1.17 [95% CI, 0.95–1.47]), episodic memory (HR, 1.11 [95% CI, 0.91–1.37]), selected components of executive function (HR, 0.95 [95% CI, 0.77–1.17]) and verbal fluency (HR, 1.18 [95% CI, 0.95–1.46]).

**Table 2:** Adjusted HRs for Incident Stroke Across Tertiles of Composite Cognitive Scores

Cognitive domain	Tertiles of composite cognitive scores						*P* for trend†
	High		Middle		Low		
	n/N	Adjusted HR (95% CI)*	n/N	Adjusted HR (95% CI)*	n/N	Adjusted HR (95% CI)*	
Global cognition							
Total	158/549	1 (reference)	179/549	1.03 (0.83–1.28)	212/549	1.17 (0.95–1.47)	0.349
Age 60–69 y	44/245	1 (reference)	82/245	1.54 (1.06–2.24)	119/245	1.86 (1.27–2.69)	0.007
Age 70–79 y	36/96	1 (reference)	25/96	0.58 (0.33–0.99)	35/96	0.94 (0.56–1.59)	0.948
Age ≥80 y	78/208	1 (reference)	72/208	0.95 (0.68–1.32)	58/208	0.88 (0.60–1.28)	0.793
Episodic memory							
Total	180/548	1 (reference)	158/548	0.81 (0.65–1.01)	210/548	1.11 (0.91–1.37)	0.487
Age 60–69 y	57/242	1 (reference)	74/242	1.18 (0.83–1.67)	111/242	1.68 (1.21–2.33)	0.007
Age 70–79 y	37/95	1 (reference)	24/95	0.58 (0.34–0.99)	34/95	1.09 (0.62–1.64)	0.978
Age ≥80 y	86/211	1 (reference)	60/211	0.65 (0.46–0.91)	65/211	0.83 (0.59–1.16)	0.487
Perceptual speed							
Total	158/549	1 (reference)	147/549	0.84 (0.67–1.05)	244/549	1.40 (1.13–1.72)	0.006
Age 60–69 y	55/248	1 (reference)	56/248	0.79 (0.54–1.15)	137/248	1.66 (1.19–2.32)	0.006
Age 70–79 y	31/95	1 (reference)	30/95	0.89 (0.52–1.52)	34/95	1.15 (0.67–1.97)	0.793
Age ≥80 y	72/206	1 (reference)	61/206	0.89 (0.62–1.26)	73/206	1.38 (0.95–2.02)	0.349
Selected components of executive function							
Total	182/557	1 (reference)	189/557	1.00 (0.82–1.23)	186/557	0.95 (0.77–1.17)	0.793
Age 60–69 y	73/249	1 (reference)	80/249	1.01 (0.73–1.39)	96/249	1.05 (0.77–1.44)	0.887
Age 70–79 y	31/97	1 (reference)	40/97	1.18 (0.72–1.91)	26/97	0.83 (0.48–1.44)	0.793
Age ≥80 y	78/211	1 (reference)	69/211	0.93 (0.67–1.30)	64/211	0.92 (0.65–1.30)	0.793
Verbal fluency							
Total	163/555	1 (reference)	193/555	1.12 (0.91–1.38)	199/555	1.18 (0.95–1.46)	0.349
Age 60–69 y	61/243	1 (reference)	77/243	1.08 (0.77–1.52)	105/243	1.31 (0.94–1.82)	0.349
Age 70–79 y	34/97	1 (reference)	33/97	0.85 (0.51–1.42)	30/97	0.93 (0.55–1.59)	0.887
Age ≥80 y	68/215	1 (reference)	83/215	1.34 (0.96–1.85)	64/215	1.21 (0.84–1.74)	0.487

HR indicates hazard ratio.

* Adjusted for age, sex, years of education, body mass index, physical inactivity, current smoking, alcohol consumption, diabetes, systolic blood pressure, antihypertensive treatment, non–high‐density lipoprotein cholesterol, cholesterol‐lowering therapy, ischemic heart disease, and atrial fibrillation.

^†^ A significant change in risk when stepping down 1 tertile is marked by a *P* for trend <0.05. *P* for trend was adjusted for false discovery rate by the Benjamini–Hochberg‐method.

The results of the age stratified analyses are shown in Table 2. Participants aged 60 to 69 years had a mean follow‐up time of 14.2 years, during which 252 of the 3134 participants had a stroke (Table S1). The mean follow‐up time was 12.1 years for the participants aged 70 to 79 years, during which 97 of the 507 participants had a stroke. Furthermore, the participants aged ≥80 years had a mean follow‐up time of 7.4 years, in which 223 of the 1271 participants had a stroke. Minimum detectable HRs given the number of events for each age strata are found in Table S2.

For global cognition, the participants aged 60 to 69 years in the low tertile had a higher risk of stroke compared with the high tertile in the fully adjusted model (HR, 1.86 [95% CI, 1.27–2.69]). The unadjusted cumulative incidence of stroke for the global cognition tertiles of participants aged 60 to 69 years is illustrated in Figure 2.

**Figure 2:**
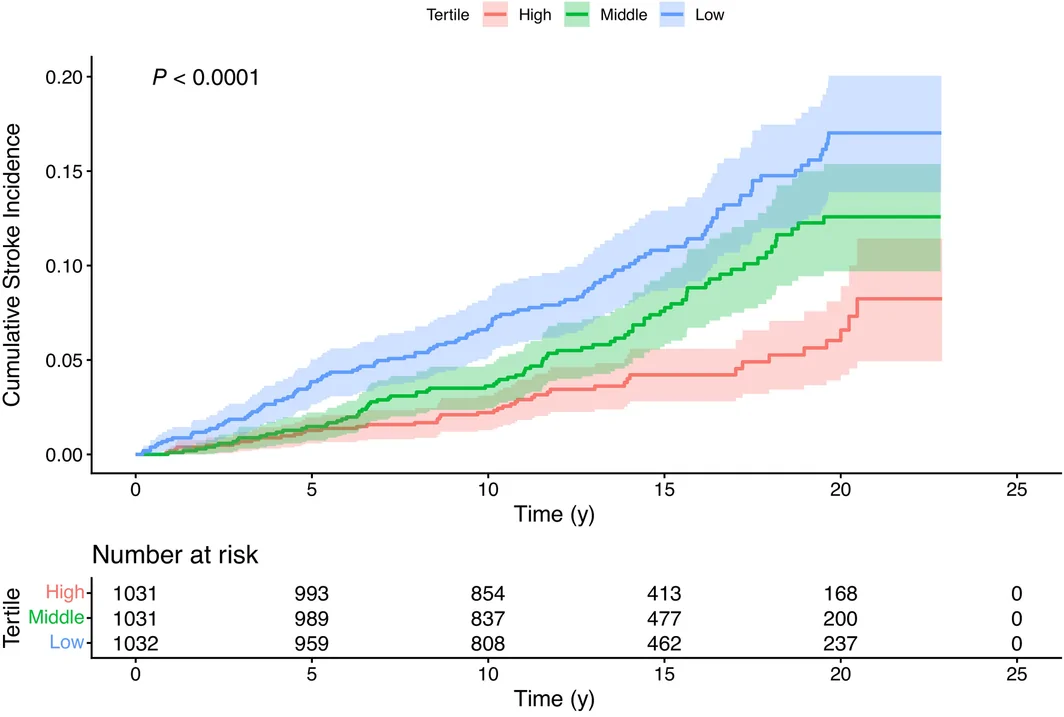
Kaplan–Meier estimates of unadjusted cumulative incidence of stroke for the 3 tertiles of global cognition of participants aged 60 to 69 years. The study population consisted of 3094 participants. Difference in cumulative incidence between tertiles analyzed by log‐rank test (*P*<0.0001). The low tertile represents participants with lower cognitive function.

In addition, for those participants aged 60 to 69 years, the risk of stroke was higher for the low tertile of episodic memory (HR, 1.68 [95% CI, 1.21–2.33]) and for the low tertile of perceptual speed (HR, 1.66 [95% CI, 1.19–2.32]) compared with the high tertile in the fully adjusted model. After adjustments for demographic factors, an increased risk of stroke was observed for participants with impairment in verbal fluency, although with nonsignificant *P* for trend (data not shown). For the low tertile of the selected components of executive function, no significant increased risk of stroke was found when compared with the high tertile (HR, 1.05 [95% CI, 0.77–1.17]).

Higher risk of stroke was not observed across cognitive domains for either the participants aged 70 to 79 or ≥80 years when comparing the low tertile with the high tertile in the fully adjusted models. No associations were found for the low tertile of perceptual speed for participants aged 70 to 79 years across models. However, for participants aged ≥80 years, higher risk of stroke was found for the low tertile of perceptual speed in model 1 (HR, 1.47 [95% CI, 1.02–2.14]) and model 2 (HR, 1.46 [95% CI, 1.00–2.11]), although with *P* for trends >0.05. Nonetheless, the association was attenuated further in model 3 (HR, 1.38 [95% CI, 0.95–2.02]).

The unadjusted HRs for the tertiles of global cognition for the total population and age subgroups are found in Table S3.

### Sensitivity Analyses With the Middle Tertile as Reference

Sensitivity analysis on the whole sample, with the middle tertile as reference, revealed higher risk of stroke for the lower tertile of global cognition compared with the middle tertile after adjustments for demographic data (data not shown). This risk was attenuated in further models of adjustment. Furthermore, in the fully adjusted models, an increased risk of stroke for the low tertile of episodic memory (HR, 1.37 [95% CI, 1.12–1.69]) and perceptual speed (HR, 1.66 [95% CI, 1.35–2.05]) were found, although the *P* for trend for episodic memory was >0.05 (Table S4). No increase in stroke risk was found for the low tertile compared with the middle tertile across models for verbal fluency (HR, 1.05 [95% CI, 0.86–1.28]) and the selected components of executive function (HR, 0.94 [95% CI, 0.77–1.16]).

In the sensitivity analysis using the middle tertile as a reference for participants aged 60 to 69 years, the low tertile of episodic memory (HR, 1.42 [95% CI, 1.06–1.91]) and perceptual speed (HR, 2.10 [95% CI, 1.53–2.89]) had a higher risk of stroke in the fully adjusted models (Table S4). No other associations were found when comparing the middle tertile with the low tertile for the age strata.

### Sex Differences

In Table 3, the results of the sensitivity analysis on women and men aged 60 to 69 years are shown, and unadjusted results are found in Table S5. Similar to the results for the whole cohort aged 60 to 69 years, there were no significant associations between the low compared with the high tertile and incident stroke in the fully adjusted models for either men or women in measures of verbal fluency or executive function.

**Table 3:** Adjusted HRs for Incident Stroke Across Tertiles of Composite Cognitive Scores, Stratified by Age Group (60–69 Years) and Sex

Cognitive domain	Tertiles of composite cognitive scores						*P* for trend†
	High		Middle		Low		
	n/N	Adjusted HR (95% CI)*	n/N	Adjusted HR (95% CI)*	n/N	Adjusted HR (95% CI)*	
Global cognition							
Women	21/106	1 (reference)	39/106	1.60 (0.93–2.75)	46/106	1.59 (0.91–2.75)	0.136
Men	23/139	1 (reference)	43/139	1.49 (0.89–2.50)	73/139	2.11 (1.28–3.47)	0.003
Episodic memory							
Women	37/105	1 (reference)	32/105	0.98 (0.60–1.59)	36/105	1.27 (0.79–2.03)	0.325
Men	20/137	1 (reference)	42/137	1.51 (0.88–2.59)	75/137	2.30 (1.39–3.81)	<0.001
Perceptual speed							
Women	26/107	1 (reference)	20/107	0.62 (0.34–1.12)	61/107	1.60 (0.98–2.60)	0.015
Men	29/141	1 (reference)	36/141	0.92 (0.56–1.51)	76/141	1.77 (1.12–2.80)	0.006
Selected components of executive function							
Women	23/107	1 (reference)	38/107	1.44 (0.86–2.43)	46/107	1.34 (0.80–2.25)	0.333
Men	50/142	1 (reference)	42/142	0.82 (0.54–1.25)	50/142	0.94 (0.62–1.40)	0.738
Verbal fluency							
Women	25/107	1 (reference)	34/107	1.11 (0.65–1.88)	48/107	1.34 (0.80–2.26)	0.253
Men	36/136	1 (reference)	43/136	1.05 (0.67–1.64)	57/136	1.25 (0.81–1.94)	0.316

HR indicates hazard ratio.

* Adjusted for age, sex, years of education, body mass index, physical inactivity, current smoking, alcohol consumption, diabetes, systolic blood pressure, antihypertensive treatment, non–high‐density lipoprotein cholesterol, cholesterol‐lowering therapy, ischemic heart disease, and atrial fibrillation.

^†^ A significant change in risk when stepping down 1 tertile is marked by a *P* for trend <0.05.

Men in the low tertile of global cognition (HR, 2.11 [95% CI, 1.28–3.47]), episodic memory (HR, 2.30 [95% CI, 1.39–3.81]) and perceptual speed (HR, 1.77 [95% CI, 1.12–2.80]) had a higher risk of stroke compared with the high tertile in the fully adjusted models. Furthermore, higher risk of stroke for men in the low compared with the high tertile of verbal fluency was observed until adjustments for lifestyle factors. No significant associations were found for women in the low tertile of global cognition (HR, 1.59 [95% CI, 0.91–2.75]), episodic memory (HR, 1.27 [95% CI, 0.79–2.03]) or perceptual speed (HR, 1.60 [95% CI, 0.98–2.60]) in the fully adjusted models. However, higher risk of stroke for the low tertile of perceptual speed remained until the fully adjusted models, similarly for episodic memory until adjustments of lifestyle factors.

### Post Hoc Competing Risk Analyses

Compared to primary analyses where an association for perceptual speed was observed, no association was found for the whole sample in the fully adjusted competing risk models (HR, 1.22 [95% CI, 0.99–1.42]; Table S6), although *P* for trend remained significant. Results for participants aged 60 to 69 years were confirmed in competing risk models. The direction of the results of the primary analyses remained in competing risk models, with the exception of the higher risk of stroke for participants aged ≥80 years in the low tertile of perceptual speed in model 2 that was not confirmed in this sensitivity analysis.

## Discussion

This study, like others, showed that older adults with low cognitive function have a higher risk of first‐time stroke in comparison with those with high cognitive function.^10^, ^11^, ^16^, ^20^ These findings were primarily seen for the youngest old, and in men, where the associated risk of those with lower cognitive functioning was 1.66 to 2.30 times the risk of those with higher cognitive functioning depending on the measured cognitive domain. Our results were independent of behavioral, psychological, and physiological factors and adds new insights on age‐ and sex‐specific associations of stroke among those with lower cognitive functioning.

Similar to the results of this study, both meta‐analyses and other recent studies have shown that impairments in global cognition are associated with higher risk of stroke.^10^, ^11^, ^16^, ^20^ However, studies that focus on impairment across several cognitive domains are few and inconsistent.^12^, ^13^, ^14^, ^15^, ^16^, ^17^, ^18^, ^19^ Prior studies showed no association between risk of all‐type stroke,^16^, ^18^ ischemic stroke,^13^, ^16^ or hemorrhagic stroke^16^ for individuals who perform worse on verbal fluency tests, which is consistent with the findings in the present study. However, studies have shown increased risk of fatal^12^ and unspecified stroke^16^ for these individuals, which suggests that associations for verbal fluency should be further investigated.

The main results, which were confirmed by sensitivity analyses, showed that older adults with lower scores on perceptual speed tests had a higher risk of stroke, which is consistent with the results of some previous studies^16^, ^17^, ^19^ but not all.^12^, ^13^, ^18^ Inconsistencies might be partially attributed to methodological differences, including the use of composite scores derived from multiple cognitive assessments rather than a single test, as well as variations in the specific test used for measurement.

Methodological differences may also account for the inconsistent findings on executive function, as the present study did not observe an increased risk of stroke among individuals with the lowest scores on selected executive function measures.^16^, ^17^, ^18^, ^19^ For instance, this study primarily measured working memory and cognitive flexibility, whereas others focus on other aspects of executive functioning such as planning, reasoning, and stronger measurements of inhibition.^16^, ^17^ Moreover, in the present study, there were several missing values for Trail Making Test B for participants who did not complete the test, as well as those who refused to participate, which might result in a nonresponse bias.

Some studies report an increased risk of stroke for individuals with impairments in episodic memory.^14^, ^15^, ^16^ These studies all included participants aged <70 years. However, in studies with samples aged >70 years were examined, these associations for episodic memory were not found.^12^, ^17^ Together with our results, this indicates that associated risk for stroke among those with impairments in memory is apparent for the younger older adults. Nonetheless, even though participants aged <70 years were included, Weinstein et al^18^ showed no association between impairment in episodic memory and stroke risk. However, age stratified analysis was not performed in that study.^18^ Adding on, de Moraes et al investigated a study population aged 48 to 67 years and failed to find an association, although power issues may have existed.^13^, ^16^

Furthermore, the differences in risk between age groups in this study may also reflect attrition bias. The older participants had shorter follow‐up periods due to a higher number of deaths from other causes. Results from competing risk models had little impact on the results for participants aged 60 to 69 years, and the direction of the results for the other groups remained largely unchanged. However, the higher risk of stroke in the main analyses for participants aged ≥80 years in the low tertile of perceptual speed until adjustments for vascular factors did not remain in competing risk analysis.

Sensitivity analysis also revealed sex‐specific associations, where heightened stroke risk was found for men aged 60 to 69 years in the low tertile of global cognition, episodic memory, and perceptual speed. Prior research has suggested that sex differences influence the relationship between vascular risk factors and stroke.^21^ It is therefore plausible that similar sex differences exist in the link between cognitive impairment and stroke risk. Proposed explanations include genetic factors as well as differences in the management of vascular conditions.^21^

Nonetheless, in contradiction to our results, Qiao et al presented a higher HR for stroke for women with cognitive impairment.^20^ However, the HRs for women in the low tertiles of global cognition and perceptual speed indicate that there might be a higher HR for stroke, even though results were nonsignificant in our study. Furthermore, Qiao et al were not able to show significant sex differences when adding an interaction term between sex and cognitive impairment.^20^ Hence, further studies exploring sex differences and possible underlying mechanisms are warranted.

In the present study, the low tertile of each domain represented those with low cognitive functioning.^17^ Notably, dividing cognitive test scores into tertiles is a robust measure of level of cognitive functioning, with some degree of misclassification. It is likely that cases of mild cognitive impairment and prodromal dementia will be present in the low tertile. While some studies adjusted for apolipoprotein E4, these data were not available for this study. Hence, it was not taken into consideration that some participants have an increased risk of amyloid angiopathy and Alzheimer dementia, which in turn affects both memory and hemorrhagic stroke risk.^33^, ^34^ Brief post hoc analysis showed more incident dementia cases in the low tertile of global cognition than the high tertile (6.2% versus 1.6%) and the stroke group compared with the nonstroke group (12.7% versus 3.0%) for participants aged 60 to 69 years, which had the longest follow‐up.

Shared risk factors between stroke and cognitive impairment may explain some of the outcomes, as associations for some domains were attenuated during the stepwise adjustments. However, the risk of stroke across impairments in domains remained even after full adjustments among the youngest participants, suggesting the influence of unmeasured factors or alternative mechanisms. One such unmeasured factor could be poor compliance with medical treatment, where those with low cognitive functioning may have poorer compliance than those in the higher cognitive functioning groups.

Given differences in risk profiles, incidence, and pathogenesis, future research could benefit from stratifying analyses by stroke subtypes, as done in former studies.^16^, ^19^ On inspection of stroke subtypes across the whole sample and age strata, the risk associations were confirmed for those with ischemic stroke, but not for hemorrhagic stroke (data not shown). Notably, as in other studies,^16^, ^19^ hemorrhagic stroke events were few, especially in each age strata. Therefore, future studies with more hemorrhagic stroke cases are needed to confirm these results.

Others have discussed that the heightened risk of stroke for those with lower cognitive functioning could be due to previous silent cerebrovascular events,^12^, ^14^, ^16^, ^17^, ^18^ as this is a suggested risk factor of both stroke and cognitive impairment.^6^, ^7^, ^8^, ^9^ Hence, the participants in the lowest tertiles might have sustained more of these events than those in the high tertiles. Since a recent magnetic resonance imaging study on GÅS data showed that >70% of those aged ≥70 years have had cerebrovascular small‐vessel disease,^35^ it might be that silent cerebrovascular events serve as a better predictor of future stroke for a younger population. For the younger population, the events are rarer, while the older population also have an increasing accumulation of risk factors, diseases, and death unrelated to stroke that might overshadow associations. This might also partly explain why the middle tertile of episodic memory for individuals aged 80 years appears to have a reduced associated risk of stroke in Cox regression, while the *P* for trend suggests no pattern in the association.

To the authors' knowledge, no study has described magnetic resonance imaging changes among those with lower cognitive function in relation to the risk of future stroke, although Weinstein et al^18^ showed that silent cerebral infarcts could not explain why those with worse performance on Trail Making Test B had a higher risk of stroke. Future studies should explore if the site of possible previous silent cerebrovascular events or the later stroke is linked to the brain area mostly associated with a particular cognitive domain. For instance, it would be valuable to investigate whether individuals with poorer performance on perceptual speed tasks already exhibit structural or functional abnormalities in domain‐relevant brain regions, such as the white matter in the parietal lobes.^36^

Strengths of this study are the use of a population‐based design, a large cohort with long follow‐up, and an extensive cognitive battery. Although no information on the characteristics of nonparticipants was available, it is likely that individuals attending GÅS are generally healthier, which may limit the generalizability of the findings. To mitigate this potential bias, home visits were offered to more fragile individuals. Moreover, the baseline cohorts had relatively high participation rates (60%–70%). Other strengths are the adjustments for multiple health covariates and the use of registry data, which prevent loss to follow‐up and recall bias.

This study is not without limitations. First, results could be underestimated due to missing data in cognitive testing, and some loss of information is expected since cognitive results were categorized into tertiles to facilitate interpretation. Second, in sex and age strata 70 to 79 years, analyses of specific cognitive domains were based on a limited number of events, which reduced statistical power. Moreover, the fully adjusted models included a large number of covariates, introducing a potential risk of overfitting in some strata. Therefore, results based on small numbers of events should be interpreted with caution. Nevertheless, there were few instances in which adjustment for covariates altered the significance levels, suggesting that the findings are relatively robust. However, future studies could possibly benefit from using more flexible methods to model age‐specific associations, as some loss of information is expected with categorization. Finally, by excluding TIA cases from the outcome, there is a risk that some misclassified cases of minor stroke were missed as computed tomographic scan was the usual imaging modality for stroke in Sweden during a significant part of the study period. Consequences could be both lower power and risk of missing effects and noneffects.

### Conclusions

The results of this study show an association between lower cognitive function in global cognition, episodic memory, and perceptual speed in younger older adults and incident stroke. Furthermore, it suggests that there are sex differences in these associations. Cognitive testing might be an important screening tool for future cerebrovascular events for this age group. More studies in the area, including magnetic resonance imaging data, analysis of stroke subtypes, and analyses on age and sex differences are needed.

## Sources of Funding

The project Good Aging in Skåne, part of the Swedish National Study on Aging and Care (www.snac.org), was supported by the Swedish Ministry of Health and Social Affairs, the County of Region Skåne, and the Swedish Research Council under Grant Number 2017‐01613; 521‐2013‐8604, 2021‐00178, and 2021‐01437. This research received no specific grant from any other funding agency in the public, commercial, or not‐for‐profit sectors.

## Disclosures

None.

## Supporting information

Tables S1–S6

STROBE Checklist
