# Supplementary material for: Lower Cognitive Function in People Aged 60 to 69 Years Is Associated With Future Stroke
Source: J Am Heart Assoc. 2026 Sep 30;15(20):e048423. doi: 10.1161/JAHA.125.048423 (PMC13769652; doi:10.1161/JAHA.125.048423)
Supplement: Supplementary file 2 [file JAH3-15-e048423-s002.zip › Supplemental Material JAHA 2025 048423 R2.docx]

# Supplemental Material

Table S1 Stroke Incidence Rate for Whole Study Population, Subgroups and the Global Cognition Tertiles

Table S2 Minimum Detectable Hazard Ratio for Strata in Analyses

Table S3 Unadjusted Hazard Ratios for Incident Stroke Across Tertiles of Composite Cognitive Scores

Table S4 Sensitivity Analysis: Adjusted Hazard Ratios for Incident Stroke Across Tertiles of Composite Cognitive Scores

Table S5 Unadjusted Hazard Ratios for Incident Stroke Across Tertiles of Composite Cognitive Scores, Stratified by Age Group (60-69 Years) and Sex

Table S6 Adjusted Subdistribution Hazard Ratios for Incident Stroke Across Tertiles of Composite Cognitive Scores

**Table S1** **Stroke Incidence Rate for Whole Study Population, Subgroups and the Global Cognition Tertiles**

| **Subgroup** |  |  |  | **Tertiles of global cognition composite cognitive scores** | | | | | | | | |
| --- | --- | --- | --- | --- | --- | --- | --- | --- | --- | --- | --- | --- |
|  | **Total** | | | **High** | | | **Middle** | | | **Low** | | |
|  | **Avg. FU, y** | **n/N** | **IR/1000 PY** | **Avg. FU, y** | **n/N** | **IR/1000 PY** | **Avg. FU, y** | **n/N** | **IR/1000 PY** | **Avg. FU, y** | **n/N** | **IR/1000 PY** |
| **All** | 12.3 | 572/4912 | 9.5 | 12.6 | 158/549 | 7.9 | 12.6 | 179/549 | 8.9 | 11.9 | 212/549 | 11.2 |
| **Age group, y/o** | | | | | | | | | | | | |
| **60-69** | 14.2 | 252/3134 | 5.7 | 14.2 | 44/245 | 3.0 | 14.4 | 82/245 | 5.5 | 14.2 | 119/245 | 8.1 |
| **70-79** | 12.1 | 97/507 | 15.8 | 13.2 | 36/95 | 16.6 | 12.9 | 25/95 | 11.7 | 10.6 | 35/95 | 20.1 |
| **80+** | 7.4 | 223/1271 | 23.6 | 8.4 | 78/208 | 23.5 | 7.9 | 72/208 | 23.0 | 6.5 | 58/208 | 22.6 |
| **Sex, 60-69 y/o** | | | | | | | | | | | | |
| **Female** | 14.6 | 109/1606 | 4.6 | 14.4 | 21/106 | 2.5 | 14.7 | 39/106 | 5.1 | 14.8 | 46/106 | 6.5 |
| **Male** | 13.8 | 143/1528 | 6.8 | 13.7 | 23/139 | 3.8 | 14.1 | 43/139 | 6.0 | 13.7 | 73/139 | 9.6 |

Abbreviations: Avg.=Average, FU=follow up, y=years, n/N=number of events/total number of participants in tertile, IR=Incidence Rate, PY=Person-Years, y/o=years old.

**Table S2 Minimum Detectable Hazard Ratio for Strata in Analyses**

| **Population** | **Events (n)^*^** | **HR (80% power)** ^†^ |
| --- | --- | --- |
| **All** | 548-557 | 1.42-1.43 |
| **60-69-year-olds** | 242-249 | 1.68-1.69 |
| **Females** | 105-107 | 2.18-2.20 |
| **Males** | 136-142 | 1.93-1.97 |
| **70-79-year-olds** | 95-97 | 2.29-2.32 |
| **80+-year-olds** | 206-215 | 1.74-1.77 |

Abbreviations: HR=Hazard Ratio. *The domain with the lowest number of events → highest number of events. †Mimimum detectable Hazard Ratio calculated by Schoenfield formula when approximately 1/3 in each group, α=0.05, power=0.80. Lowest → Highest.

**Table S3 Unadjusted Hazard Ratios for Incident Stroke Across Tertiles of Composite Cognitive Scores**

|  | **Tertiles of composite cognitive scores** | | | | | |  |
| --- | --- | --- | --- | --- | --- | --- | --- |
|  | **High** | | **Middle** | | **Low** | |  |
| **Cognitive**  **Domain** | **n/N** | **cHR**  **(95% CI)** | **n/N** | **cHR**  **(95% CI)** | **n/N** | **cHR**  **(95% CI)** | ***p* for trend*** |
| **Global** |  |  |  |  |  |  |  |
| **Total** | 158/549 | 1 (ref) | 179/549 | 1.13 (0.91-1.39) | 212/549 | 1.41 (1.15-1.74) | 0.003 |
| **60-69 y/o** | 44/245 | 1 (ref) | 82/245 | 1.81 (1.25-2.61) | 119/245 | 2.64 (1.87-3.74) | <0.001 |
| **70-79 y/o** | 36/96 | 1 (ref) | 25/96 | 0.72 (0.43-1.20) | 35/96 | 1.28 (0.81-2.05) | 0.518 |
| **80+ y/o** | 78/208 | 1 (ref) | 72/208 | 0.98 (0.71-1.35) | 58/208 | 0.98 (0.70-1.38) | 0.997 |
|  |  |  |  |  |  |  |  |
| **EM** |  |  |  |  |  |  |  |
| **Total** | 180/548 | 1 (ref) | 158/548 | 0.88 (0.71-1.09) | 210/548 | 1.21 (0.99-1.48) | 0.104 |
| **60-69 y/o** | 57/242 | 1 (ref) | 74/242 | 1.30 (0.92-1.84) | 111/242 | 1.92 (1.40-2.65) | <0.001 |
| **70-79 y/o** | 37/95 | 1 (ref) | 24/95 | 0.68 (0.41-1.14) | 34/95 | 1.18 (0.74-1.88) | 0.740 |
| **80+ y/o** | 86/211 | 1 (ref) | 60/211 | 0.68 (0.49-0.95) | 65/211 | 0.89 (0.65-1.23) | 0.570 |
|  |  |  |  |  |  |  |  |
| **PS** |  |  |  |  |  |  |  |
| **Total** | 158/549 | 1 (ref) | 147/549 | 0.92 (0.74-1.16) | 244/549 | 1.63 (1.33-1.99) | <0.001 |
| **60-69 y/o** | 55/248 | 1 (ref) | 56/248 | 0.96 (0.66-1.39) | 137/248 | 2.34 (1.71-3.20) | <0.001 |
| **70-79 y/o** | 31/95 | 1 (ref) | 30/95 | 1.24 (0.75-2.06) | 34/95 | 1.68 (1.03-2.75) | 0.085**^†^** |
| **80+ y/o** | 72/206 | 1 (ref) | 61/206 | 0.88 (0.63-1.24) | 73/206 | 1.50 (1.08-2.08) | 0.058**^†^** |
|  |  |  |  |  |  |  |  |
| **EXC** |  |  |  |  |  |  |  |
| **Total** | 182/557 | 1 (ref) | 189/557 | 1.05 (0.86-1.29) | 186/557 | 1.06 (0.86-1.30) | 0.743 |
| **60-69 y/o** | 73/249 | 1 (ref) | 80/249 | 1.11 (0.81-1.52) | 96/249 | 1.32 (0.97-1.78) | 0.136 |
| **70-79 y/o** | 31/97 | 1 (ref) | 40/97 | 1.29 (0.81-2.06) | 26/97 | 0.98 (0.58-1.66) | 0.997 |
| **80+ y/o** | 78/211 | 1 (ref) | 69/211 | 0.91 (0.66-1.26) | 64/211 | 0.93 (0.67-1.30) | 0.792 |
|  |  |  |  |  |  |  |  |
| **VF** |  |  |  |  |  |  |  |
| **Total** | 163/555 | 1 (ref) | 193/555 | 1.19 (0.96-1.46) | 199/555 | 1.29 (1.05-1.59) | 0.043 |
| **60-69 y/o** | 61/243 | 1 (ref) | 77/243 | 1.24 (0.89-1.74) | 105/243 | 1.73 (1.26-2.37) | 0.002 |
| **70-79 y/o** | 34/97 | 1 (ref) | 33/97 | 0.96 (0.60-1.56) | 30/97 | 1.02 (0.62-1.67) | 0.997 |
| **80+ y/o** | 68/215 | 1 (ref) | 83/215 | 1.31 (0.95-1.81) | 64/215 | 1.25 (0.89-1.76) | 0.298 |

Abbreviations: n/N=number of events/total number of participants in tertile, cHR=crude Hazard Ratio, CI=Confidence Interval, Global=Global Cognition, EM=Episodic Memory, PS=Perceptual Speed, EXC=selected components of Executive Function, VF=Verbal Fluency, y/o=years old, ref=reference group. *A significant change in risk when stepping down one tertile is marked by a *p* for trend<0.05. *p* for trend was adjusted for false discovery rate by the Benjamini–Hochberg-method. **†** Attenuated in Benjamini-Hochberg-correction.

**Table S4 Sensitivity Analysis: Adjusted Hazard Ratios for Incident Stroke Across Tertiles of Composite Cognitive Scores**

|  | **Tertiles of composite cognitive scores** | | | | | |  |
| --- | --- | --- | --- | --- | --- | --- | --- |
|  | **High** | | **Middle** | | **Low** | |  |
| **Cognitive**  **Domain** | **n/N** | **aHR**  **(95% CI)^*^** | **n/N** | **aHR**  **(95% CI)^*^** | **n/N** | **aHR**  **(95% CI)^*^** | ***p* for trend^†^** |
| **Global** |  |  |  |  |  |  |  |
| **Total** | 158/549 | 0.97 (0.78-1.21) | 179/549 | 1 (ref) | 212/549 | 1.15 (0.94-1.40) | 0.123 |
| **60-69 y/o** | 44/245 | 0.65 (0.44-0.94) | 82/245 | 1 (ref) | 119/245 | 1.20 (0.90-1.60) | 0.001 |
| **70-79 y/o** | 36/96 | 1.74 (1.01-3.00) | 25/96 | 1 (ref) | 35/96 | 1.63 (0.96-2.75) | 0.900 |
| **80+ y/o** | 78/208 | 1.05 (0.75-1.47) | 72/208 | 1 (ref) | 58/208 | 0.92 (0.64-1.32) | 0.515 |
|  |  |  |  |  |  |  |  |
| **EM** |  |  |  |  |  |  |  |
| **Total** | 180/548 | 1.23 (0.99-1.53) | 158/548 | 1 (ref) | 210/548 | 1.37 (1.12-1.69) | 0.247 |
| **60-69 y/o** | 57/242 | 0.85 (0.60-1.20) | 74/242 | 1 (ref) | 111/242 | 1.42 (1.06-1.91) | 0.001 |
| **70-79 y/o** | 37/95 | 1.72 (1.00-2.93) | 24/95 | 1 (ref) | 34/95 | 1.72 (1.00-2.94) | 0.978 |
| **80+ y/o** | 86/211 | 1.53 (1.10-2.15) | 60/211 | 1 (ref) | 65/211 | 1.27 (0.89-1.82) | 0.238 |
|  |  |  |  |  |  |  |  |
| **PS** |  |  |  |  |  |  |  |
| **Total** | 158/549 | 1.19 (0.95-1.49) | 147/549 | 1 (ref) | 244/549 | 1.66 (1.35-2.05) | <0.001 |
| **60-69 y/o** | 55/248 | 1.27 (0.87-1.86) | 56/248 | 1 (ref) | 137/248 | 2.10 (1.53-2.89) | <0.001 |
| **70-79 y/o** | 31/95 | 1.13 (0.66-1.92) | 30/95 | 1 (ref) | 34/95 | 1.28 (0.78-2.13) | 0.578 |
| **80+ y/o** | 72/206 | 1.13 (0.79-1.61) | 61/206 | 1 (ref) | 73/206 | 1.56 (1.08-2.24) | 0.118 |
|  |  |  |  |  |  |  |  |
| **EXC** |  |  |  |  |  |  |  |
| **Total** | 182/557 | 0.99 (0.81-1.22) | 189/557 | 1 (ref) | 186/557 | 0.94 (0.77-1.16) | 0.634 |
| **60-69 y/o** | 73/249 | 0.99 (0.72-1.37) | 80/249 | 1 (ref) | 96/249 | 1.04 (0.77-1.40) | 0.773 |
| **70-79 y/o** | 31/97 | 0.85 (0.52-1.38) | 40/97 | 1 (ref) | 26/97 | 0.70 (0.42-1.17) | 0.534 |
| **80+ y/o** | 78/211 | 1.07 (0.77-1.49) | 69/211 | 1 (ref) | 64/211 | 0.98 (0.69-1.39) | 0.622 |
|  |  |  |  |  |  |  |  |
| **VF** |  |  |  |  |  |  |  |
| **Total** | 163/555 | 0.89 (0.72-1.10) | 193/555 | 1 (ref) | 199/555 | 1.05 (0.86-1.28) | 0.268 |
| **60-69 y/o** | 61/243 | 0.92 (0.66-1.30) | 77/243 | 1 (ref) | 105/243 | 1.20 (0.89-1.62) | 0.105 |
| **70-79 y/o** | 34/97 | 1.17 (0.71-1.95) | 33/97 | 1 (ref) | 30/97 | 1.08 (0.66-1.80) | 0.798 |
| **80+ y/o** | 68/215 | 0.75 (0.54-1.04) | 83/215 | 1 (ref) | 64/215 | 0.90 (0.65-1.26) | 0.271 |

Abbreviations: n/N=number of events/total number of participants in tertile, aHR=adjusted Hazard Ratio, CI=Confidence Interval, Global=Global Cognition, EM=Episodic Memory, PS=Perceptual Speed, EXC=selected components of Executive Function, VF=Verbal Fluency, y/o=years old, ref=reference group. *Adjusted for age, sex, years of education, body mass index, physical inactivity, current smoking, use of alcohol, diabetes mellitus, systolic blood pressure, antihypertensive treatment, non-HDL-cholesterol, cholesterol-lowering treatment, ischemic heart disease and atrial fibrillation. † A significant change in risk when stepping down one tertile is marked by a *p* for trend<0.05.

**Table S5 Unadjusted Hazard Ratios for Incident Stroke Across Tertiles of Composite Cognitive Scores, Stratified by Age Group (60-69 Years) and Sex**

|  | **Tertiles of composite cognitive scores** | | | | | |  |
| --- | --- | --- | --- | --- | --- | --- | --- |
|  | **High** | | **Middle** | | **Low** | |  |
| **Cognitive**  **Domain** | **n/N** | **cHR**  **(95% CI)** | **n/N** | **cHR**  **(95% CI)** | **n/N** | **cHR**  **(95% CI)** | ***p* for trend*** |
| **Global** |  |  |  |  |  |  |  |
| **F** | 21/106 | 1 (ref) | 39/106 | 2.01 (1.18-3.42) | 46/106 | 2.56 (1.53-4.30) | <0.001 |
| **M** | 23/139 | 1 (ref) | 43/139 | 1.57 (0.95-2.61) | 73/139 | 2.50 (1.57-4.01) | <0.001 |
|  |  |  |  |  |  |  |  |
| **EM** |  |  |  |  |  |  |  |
| **F** | 37/105 | 1 (ref) | 32/105 | 1.14 (0.71-1.83) | 36/105 | 1.47 (0.93-2.33) | 0.103 |
| **M** | 20/137 | 1 (ref) | 42/137 | 1.44 (0.85-2.45) | 75/137 | 2.21 (1.35-3.62) | <0.001 |
|  |  |  |  |  |  |  |  |
| **PS** |  |  |  |  |  |  |  |
| **F** | 26/107 | 1 (ref) | 20/107 | 0.81 (0.45-1.46) | 61/107 | 2.49 (1.57-3.95) | <0.001 |
| **M** | 29/141 | 1 (ref) | 36/141 | 1.01 (0.62-1.64) | 76/141 | 2.11 (1.37-3.24) | <0.001 |
|  |  |  |  |  |  |  |  |
| **EXC** |  |  |  |  |  |  |  |
| **F** | 23/107 | 1 (ref) | 38/107 | 1.59 (0.95-2.68) | 46/107 | 1.84 (1.12-2.68) | 0.018 |
| **M** | 50/142 | 1 (ref) | 42/142 | 0.89 (0.59-1.34) | 50/142 | 1.10 (0.74-1.63) | 0.647 |
|  |  |  |  |  |  |  |  |
| **VF** |  |  |  |  |  |  |  |
| **F** | 25/107 | 1 (ref) | 34/107 | 1.38 (0.82-2.31) | 48/107 | 2.00 (1.23-3.24) | 0.004 |
| **M** | 36/136 | 1 (ref) | 43/136 | 1.13 (0.73-1.76) | 57/136 | 1.52 (1.00-2.31) | 0.043 |

Abbreviations: n/N=number of events/total number of participants in tertile, cHR=crude Hazard Ratio CI=Confidence Interval, Global=Global cognition, EM=Episodic Memory, PS=Perceptual Speed, EXC=selected components of Executive Function, VF=Verbal Fluency, F=Female, M=Male, ref=reference group.

*A significant change in risk when stepping down one tertile is marked by a *p* for trend<0.05.

**Table S6 Adjusted Subdistribution Hazard Ratios for Incident Stroke Across Tertiles of Composite Cognitive Scores**

|  | **Tertiles of composite cognitive scores** | | | | | |  |
| --- | --- | --- | --- | --- | --- | --- | --- |
|  | **High** | | **Middle** | | **Low** | |  |
| **Cognitive**  **Domain** | **n/N** | **aSHR**  **(95% CI)^*^** | **n/N** | **aSHR**  **(95% CI)^*^** | **n/N** | **aSHR**  **(95% CI)^*^** | ***p* for trend^†^** |
| **Global** |  |  |  |  |  |  |  |
| **Total** | 158/549 | 1 (ref) | 179/549 | 1.00 (0.81-1.25) | 212/549 | 1.04 (0.84-1.29) | 0.680 |
| **60-69 y/o** | 44/245 | 1 (ref) | 82/245 | 1.54 (1.06-2.24) | 119/245 | 1.83 (1.26-2.65) | 0.002 |
| **70-79 y/o** | 36/96 | 1 (ref) | 25/96 | 0.57 (0.33-0.99) | 35/96 | 0.85 (0.50-1.43) | 0.605 |
| **80+ y/o** | 78/208 | 1 (ref) | 72/208 | 0.94 (0.68-1.29) | 58/208 | 0.74 (0.51-1.07) | 0.119 |
|  |  |  |  |  |  |  |  |
| **EM** |  |  |  |  |  |  |  |
| **Total** | 180/548 | 1 (ref) | 158/548 | 0.80 (0.64-0.99) | 210/548 | 1.01 (0.83-1.25) | 0.796 |
| **60-69 y/o** | 57/242 | 1 (ref) | 74/242 | 1.18 (0.83-1.66) | 111/242 | 1.65 (1.19-2.28) | 0.002 |
| **70-79 y/o** | 37/95 | 1 (ref) | 24/95 | 0.57 (0.33-0.97) | 34/95 | 0.84 (0.50-1.40) | 0.526 |
| **80+ y/o** | 86/211 | 1 (ref) | 60/211 | 0.66 (0.47-0.92) | 65/211 | 0.75 (0.53-1.05) | 0.090 |
|  |  |  |  |  |  |  |  |
| **PS** |  |  |  |  |  |  |  |
| **Total** | 158/549 | 1 (ref) | 147/549 | 0.82 (0.65-1.02) | 244/549 | 1.22 (0.99-1.42) | 0.035 |
| **60-69 y/o** | 55/248 | 1 (ref) | 56/248 | 0.79 (0.54-1.15) | 137/248 | 1.64 (1.17-2.30) | <0.001 |
| **70-79 y/o** | 31/95 | 1 (ref) | 30/95 | 0.86 (0.50-1.47) | 34/95 | 0.96 (0.57-1.64) | 0.919 |
| **80+ y/o** | 72/206 | 1 (ref) | 61/206 | 0.85 (0.60-1.20) | 73/206 | 1.10 (0.75-1.62) | 0.671 |
|  |  |  |  |  |  |  |  |
| **EXC** |  |  |  |  |  |  |  |
| **Total** | 182/557 | 1 (ref) | 189/557 | 0.98 (0.96-1.02) | 186/557 | 0.88 (0.71-1.09) | 0.240 |
| **60-69 y/o** | 73/249 | 1 (ref) | 80/249 | 0.99 (0.71-1.36) | 96/249 | 1.03 (0.74-1.42) | 0.882 |
| **70-79 y/o** | 31/97 | 1 (ref) | 40/97 | 1.23 (0.77-1.98) | 26/97 | 0.75 (0.42-1.32) | 0.319 |
| **80+ y/o** | 78/211 | 1 (ref) | 69/211 | 0.91 (0.65-1.26) | 64/211 | 0.86 (0.61-1.22) | 0.382 |
|  |  |  |  |  |  |  |  |
| **VF** |  |  |  |  |  |  |  |
| **Total** | 163/555 | 1 (ref) | 193/555 | 1.09 (0.89-1.35) | 199/555 | 1.05 (0.84-1.29) | 0.722 |
| **60-69 y/o** | 61/243 | 1 (ref) | 77/243 | 1.09 (0.77-1.53) | 105/243 | 1.27 (0.91-1.77) | 0.156 |
| **70-79 y/o** | 34/97 | 1 (ref) | 33/97 | 0.95 (0.57-1.58) | 30/97 | 0.81 (0.48-1.38) | 0.436 |
| **80+ y/o** | 68/215 | 1 (ref) | 83/215 | 1.30 (0.94-1.80) | 64/215 | 1.04 (0.73-1.52) | 0.740 |

Abbreviations: n/N=number of events/total number of participants in tertile, aSHR=adjusted Sub distribution Hazard Ratio, CI=Confidence Interval, Global=Global Cognition, EM=Episodic Memory, PS=Perceptual Speed, EXC=selected components of Executive Function, VF=Verbal Fluency, y/o=years old, ref=reference group. * Adjusted for age, sex, years of education, body mass index, physical inactivity, current smoking, use of alcohol, diabetes mellitus, systolic blood pressure, antihypertensive treatment, non-HDL-cholesterol, cholesterol-lowering treatment, ischemic heart disease and atrial fibrillation. † A significant change in risk when stepping down one tertile is marked by a *p* for trend<0.05.
